# Seeing Pedestrian in the Dark via Multi-Task Feature Fusing-Sharing Learning for Imaging Sensors

**DOI:** 10.3390/s20205852

**Published:** 2020-10-16

**Authors:** Yuanzhi Wang, Tao Lu, Tao Zhang, Yuntao Wu

**Affiliations:** 1 Hubei Key Laboratory of Intelligent Robot, School of Computer Science and Engineering, Wuhan Institute of Technology, Wuhan 430073, China; 21907010074@wit.edu.cn (Y.W.); lut@wit.edu.cn (T.L.); ytwu@wit.edu.cn (Y.W.); 2 School of Electronics and Information Engineering, Beihang University, Beijing 100191, China

**Keywords:** multi-task learning, image relighting, pedestrian detection

## Abstract

Pedestrian detection is an essential problem of computer vision, which has achieved tremendous success under controllable conditions using visible light imaging sensors in recent years. However, most of them do not consider low-light environments which are very common in real-world applications. In this paper, we propose a novel pedestrian detection algorithm using multi-task learning to address this challenge in low-light environments. Specifically, the proposed multi-task learning method is different from the most commonly used multi-task learning method—the parameter sharing mechanism—in deep learning. We design a novel multi-task learning method with feature-level fusion and a sharing mechanism. The proposed approach contains three parts: an image relighting subnetwork, a pedestrian detection subnetwork, and a feature-level multi-task fusion learning module. The image relighting subnetwork adjusts the low-light image quality for detection, the pedestrian detection subnetwork learns enhanced features for prediction, and the feature-level multi-task fusion learning module fuses and shares features among component networks for boosting image relighting and detection performance simultaneously. Experimental results show that the proposed approach consistently and significantly improves the performance of pedestrian detection on low-light images obtained by visible light imaging sensor.

## 1. Introduction

Pedestrian detection is a vital problem in computer vision with significant impact on a number of applications, such as advanced driver assistance systems, robot navigation, and intelligent video surveillance systems. These applications use a large number of imaging sensors. The continuous development of deep learning has made a huge contribution to the performance of pedestrian detection. Generally speaking, pedestrian detection is a specific domain of object detection [1,2,3,4,5,6,7,8,9]. From this perspective, pedestrian detection algorithms in deep learning can be divided into two categories: anchor box-based and keypoint-based methods.

In pedestrian detection algorithms that involve designing handcrafted features, researchers usually need to design the complicated features to ensure the detection performance. Pierre et al. [10] proposed unsupervised multi-stage feature learning and designed low-level features to improve the performance of pedestrian detection. Shen et al. [11] proposed a novel Taylor feature transform (TAFT) feature for pedestrian detection, which is derived by treating an image by a 2D function and applying its Taylor series for approximation. Paolanti et al. [12] used multiple nearest neighbor classifiers and neighborhood component features selection to achieve pedestrian re-identification. Although the above methods have achieved good results, the heavy feature design brings a lot of resource consumption. Girshick et al. [1] first introduced Convolutional Neural Networks (CNNs) into object detection, named “R-CNN”, which enabled object detection without designing handcrafted features. Then, Girshick [2] optimized the training process for R-CNN and proposed a single-stage training algorithm that speeds up the training and improves the performance of the algorithm. Ren et al. [3] first proposed the anchor boxes-based detection algorithm and a Region Proposal Network (RPN) that shares fully image convolutional features with the detection network, which can achieve almost costless region proposals. Since then, anchor boxes have been widely used in SSD [4], YOLOv2 [5], YOLOv3 [6], and other excellent object detection algorithms. Liu et al. [13] presented an asymptotic localization fitting module to refine anchor boxes step by step into final detection results. Although the above anchor boxes-based methods achieved acceptable performance, they depended on a very large set of anchor boxes and heavy training burden.

Keypoint-based object detection algorithms generate object bounding boxes by detecting and grouping their key points. They greatly simplify the output of the network and eliminate the need for designing anchor boxes. Zhou et al. [14] modeled an object as a center point of bounding box, and this detector used keypoint estimation to find center points and regressed to all other object properties, such as size, 3D location and orientation. Liu et al. [15] proposed a Center and Scale Prediction (CSP) based detector as new perspective of treating pedestrian detection and predicting the scale of the central points. Law et al. [16] presented a novel approach, named “CornerNet”, to detect an object bounding box as a pair of keypoints, the top-left corner and the bottom-right corner. Law et al. [17] subsequently improved CornerNet and named as “CornerNet-Saccade”, which adopted the attention mechanism to further improve the performance of object detection and reduce parameters.

Although the above-mentioned pedestrian detection algorithms yield satisfactory performance under normal lighting conditions. Most of them do not consider pedestrian detection in low-light environments. As we know, in real-world applications, normal lighting condition is not always guaranteed. On the contrary, the low-light environment is very common. To address the low-light pedestrian detection problem, Kruthiventi et al. [18] proposed a deep convolutional network under low illumination conditions. However, it needs to be trained by thermal and RGB images, and the thermal images need to be obtained by infrared thermal imaging sensors, which incurs high cost. The main reason for the poor pedestrian detection performance in low-light environment is that low lighting causes serious distortion of color and texture information in the obtained inputs. However, color and texture information plays a vital role in pedestrian detection.

For both pedestrian detection and image relighting tasks, there is a basic discovery that these two tasks can share with some features during learning due to learning framework from CNNs. Inspired by multi-task learning [19], we propose a novel multi-task learning method with feature-level fusion and sharing mechanism, which will be described in Section 3. The proposed Seeing Pedestrian in the Dark via Multi-task Feature Fusing-sharing Learning, dubbed as “SPMF” in this paper, has three components: an image relighting subnetwork, a pedestrian detection subnetwork, and a feature-level multi-task fusion learning module. The first two subnetworks implement light enhancement and object detection, respectively. By cascading above two subnetworks, the feature-level multi-task fusion learning module shares fused features among different subnetworks for image relighting and detection. Experimental results show that SPMF consistently and significantly improves the accuracy of pedestrian detection on low-light images.

The contributions of this paper are summarized as follows.

(i) Different from most commonly used multi-task learning methods with a parameter sharing mechanism for deep learning, we propose a novel multi-task learning method with feature-level fusion and sharing mechanism in the serial tasks. The proposed multi-task learning method is used in the feature-level multi-task fusion learning module to fuse the feature information of upstream and downstream tasks, and then the fused features are learned for each tasks to boost upstream and downstream tasks performance simultaneously.
(ii) A novel Self-Calibrated Split Attention Block is proposed, named “SCSAB”, which combines Self-Calibrated convolution layer and Split Attention mechanism. SCSAB further improves the ability of SPMF to detect pedestrians in the dark.

## 2. Related Work

In this section, we first describe the present situation of multi-task learning and then focus on introducing two most common methods for multi-task learning in Deep Learning. Next, the self-calibrated convolution and Split Attention Networks are introduced in detail.

### 2.1. Multi-Task Learning in Deep Learning

Multi-task learning has been successful in the field of machine learning, from image processing [2] to language processing [20] and medical drug discovery [21]. There are many forms of multi-task learning: joint learning, learning to learn, and assisted task learning are only some names that have been used to refer to it. Caruana [22] summarized the merit of multi-task learning: “Multi-task learning improves generalization by leveraging the domain-specific information contained in the training signals of related tasks”.

**Figure 1:**
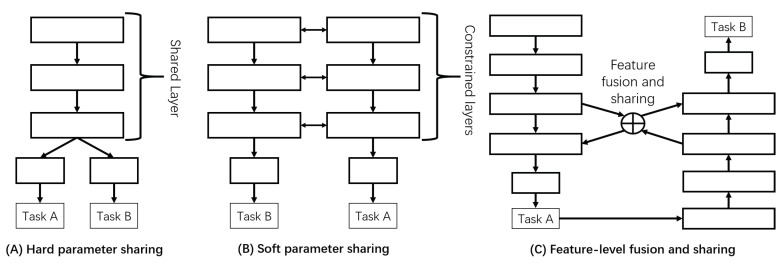
Three multi-task learning methods. (**A**,**B**) Two widely used multi-task learning methods. (**C**) The method proposed in this paper, which is a novel multi-task learning method with feature-level fusion and sharing.

In view of the great success of multi-task learning in machine learning, Ruder [19] summarized two methods of multi-task learning for Deep Learning: the hard parameter sharing method and soft parameter sharing method. Panels (A,B) in Figure 1 show the structure of these two methods. Hard parameter sharing was originally proposed by Caruana [23], but its use is still very common in recent years. Long et al. [24] proposed a Deep Relationship Networks, which was a typical representative of Hard parameter sharing, to learn multiple tasks. Although the hard parameter sharing method greatly reduces the risk of overfitting for each task, it collapse quickly if there is no close relationship between tasks or reasoning at different levels. In the soft parameter sharing method, each task has its own model and parameters. Then, the distance between model parameters is regularized to encourage the parameters to be similar. Misra et al. [25] proposed a Cross-stitch Network for multi-task learning, which used the soft parameter sharing mechanism. They used cross-stitch units allowing the model to determine how task-specific networks would use knowledge from other tasks by learning a parameterized linear combination of the output of the previous layers. Gao et al. [26] proposed a novel convolutional neural network structure for multi-task learning, named “NDDR-CNN”, which enables automatic feature fusing at every layer from different tasks. Then, the authors of [27] extended the works in [25,26], which proposed the neural architecture search (NAS) into multi-task learning. This gets rid of the way of feature fusion between different tasks of hand-crafted designing.

Although the above two multi-task learning methods are widely used in machine learning, these learning mechanisms can only be used for tasks with high relevance and parallel tasks. If these multi-task learning mechanisms are used between the upstream and downstream tasks, it may lead to poor performance, and model training may be difficult to converge. In this study, we propose a novel multi-task learning method with feature-level fusion and sharing mechanism to fuse the feature information of upstream and downstream tasks. In this way, the upstream and downstream tasks can boost performance simultaneously. Figure 1C shows the structure of this method.

### 2.2. Self-Calibrated Convolutions and Split Attention Network

In recent years, novel architecture designing has made world-renowned progress. As an early work, ResNet [28] introduces the residual connections and using batch normalization [29] to greatly improve the sequential structure, making it possible to build very deep neural networks and to enhance the performance of versatile tasks in computer vision [30,31,32,33]. ResNeXt [34] uses group convolutional layers or increases their width to extend ResNet. GoogLeNet [35] has been successful in multipath representation, in which each network block is composed of different convolutional kernels. NASNet [36] learns to automatically build model architecture by exploring a predefined search space. DenseNet [37] aggregates the features of different convolution layers through complex bottom-up skip connections. SENet [38] introduces the squeezing-and-excitation operation and channel-attention mechanism to explicitly model the inner relationship in channel-level.

The above methods focus too much on designing or adjusting the network architectures to generate rich and better feature representations, which requires a lot of manpower, material resources, and time. To solve this problem, Liu et al. [39] presents a novel Self-Calibrated Convolution that explicitly expands fields-of-view of each convolutional layer through internal Interactive and hence enriches the output features. Self-Calibrated Convolutions improve the basic convolutional feature representation process of CNNs without adjusting and designing the model architectures. Benefiting from the great contribution of ResNet and its variants in deep learning, Zhang et al. [40] proposed ResNeSt, which combines the attention mechanism of SENet [38], SKNet [41], and ResNeXt [34] into group-level. ResNeSt introduced the Split Attention Network, which enables feature-map attention across different feature-map groups. In this study, we propose a Self-Calibrated Split Attention Block, dubbed as “SCSAB” in this paper, which combines Self-Calibrated Convolutions and Split Attention Network. The details of SCSAB are explained in Section 3.

## 3. Proposed Methods

In this section, we present the architecture of the proposed network, including the main framework and details for individual modules.

### 3.1. Network Architecture

**Figure 2:**
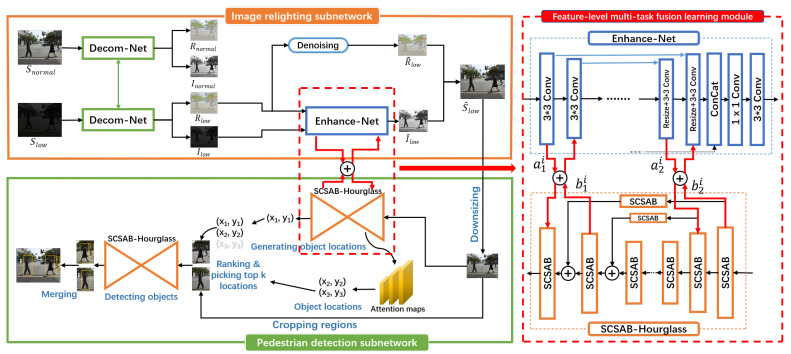
The architecture of SPMF includes three parts: image relighting subnetwork, pedestrian detection subnetwork, and feature-level multi-task fusion learning module. The feature-level multi-task fusion learning module uses feature-level fusion and sharing mechanism to fuse features from two subnetworks, and then shares fused features to these two subnetworks.

In order to boost the pedestrian detection performance in low-light environment, we use a novel multi-task learning mechanism to simultaneously learn two-task shared features for rendering rich texture images and for obtaining better pedestrian detection performance in low-light environment. The proposed approach consists of three parts: an image relighting subnetwork, a pedestrian detection subnetwork, and a feature-level multi-task fusion learning module. Figure 2 shows the architecture of SPMF, including two subnetworks and the detailed structure of feature-level multi-task fusion learning module.

### 3.2. Self-Calibrated Split Attention Block (SCSAB)

**Figure 3:**
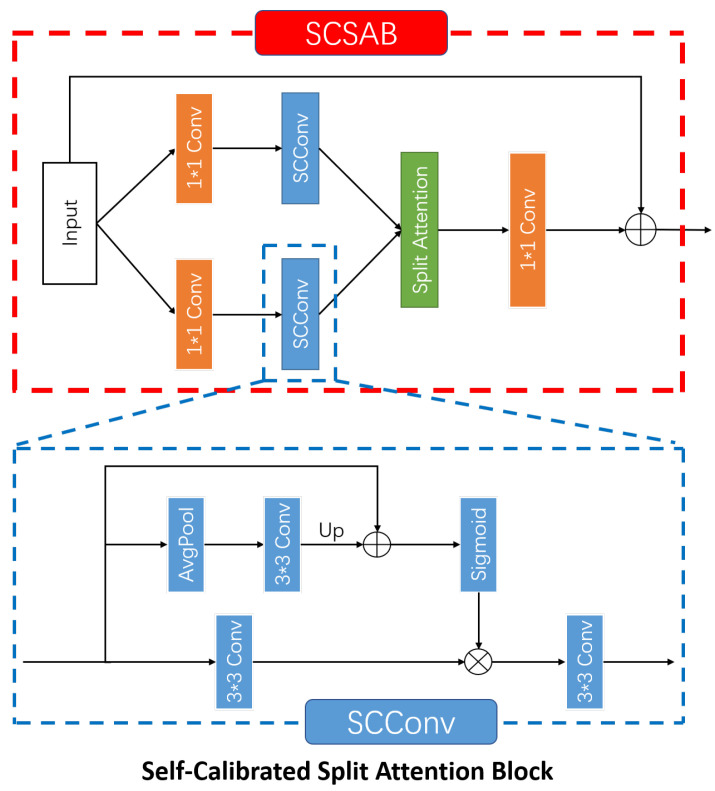
Architecture of SCSAB. AvgPool layer is used to produce a small scale space and Up is a bilinear interpolation operator that maps the small size feature space to the original size feature space.

Previous CNN-based object/pedestrian detection methods [14,15,16,17] use large-scale residual blocks, which improve the performance of the detection algorithm. However, in the convolution feature transformation, the residual blocks cannot obtain a larger fields-of-view for each spatial position. To efficiently fuse informative contextual information for each spatial location and expand receptive field of each convolutional layer, we propose the Self-Calibrated Split Attention Block (SCSAB), which combines Self-Calibrated Convolutions [39] and Split Attention Network [40]. The detailed structure of SCSAB is shown in Figure 3. SCSAB can perform convolutional feature transformation in two different feature spaces: an original size feature space with the same resolution of the feature map and the input, and a small size feature space after downsampling. Where the SCConv is the Self-Calibrated Convolution layer, and the detailed structure of Split Attention is proposed in Section 3 in [40].

### 3.3. Multi-Task Learning with Feature-Level Fusion and Sharing

In this subsection, we introduce a novel multi-task learning method with a feature-level fusion and sharing mechanism and explain in detail how it works.

Different from most commonly used multi-task learning methods of parameter sharing mechanism in deep learning, the proposed multi-task learning method with feature-level fusion and sharing mechanism is used to fuse the feature information of upstream and downstream tasks, and then the fused features are learned for upstream and downstream tasks simultaneously.

**Figure 4:**
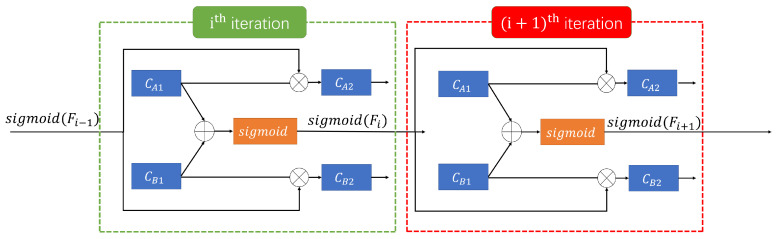
The mechanism of multi-task learning method with feature-level fusion and sharing.

The workflow of the proposed mechanism is shown in Figure 4. Suppose there are two tasks, A and B, task A is an upstream task and task B is a downstream task. The output feature of the convolutional layer $C_{A1}$ in the task A is $O_{C_{A1}}$, and the output feature of the convolutional layer $C_{B1}$ in the task B is $O_{C_{B1}}$, and $C_{A2}$ and $C_{B2}$ are the next convolutional layers of $C_{A1}$ and $C_{B1}$, respectively. $I_{C_{A2}}$ is the input of $C_{A2}$ convolutional layer, $I_{C_{B2}}$ is the input of $C_{B2}$ convolutional layer, and $F_{i}$ is the fused feature obtained from the $i^{th}$ end-to-end alternately iteration. The $F_{i}$ is formulated as

(1) $$F_{i}=\left(O_{C_{A1}}+O_{C_{B1}}\right)/2.$$

When i=1, there is no fused feature between the task *A* and task *B*. Here, the $I_{C_{A2}}$ and $I_{C_{B2}}$ come from the output of $C_{A1}$ and $C_{B1}$. $I_{C_{A2}}$ and $I_{C_{B2}}$ are formulated as

(2) $$\left\{\begin{array}{c} I_{C_{A2}}=O_{C_{A1}}\\ I_{C_{B2}}=O_{C_{B1}} \end{array}\right..$$

When i>1, the $i^{th}$ iteration for $I_{C_{A2}}$ and $I_{C_{B2}}$ are formulated as

(3) $$\left\{\begin{array}{c} \left.I_{C_{A2}}=O_{C_{A1}}\times {sigmoid\left(F\right.}_{i-1}\right)\\ I_{C_{B2}}=O_{C_{B1}}\times sigmoid\left(F_{i-1}\right) \end{array}\right.,$$

where $F_{i-1}$ is produced in ${\left(i-1\right)}^{th}$ iteration, then is mapped by Sigmoid function, and finally is multiplied by $O_{C_{A1}}$ and $O_{C_{B1}}$, respectively, as the input of $C_{A2}$ and $C_{B2}$.

### 3.4. Image Relighting Subnetwork

In the image relighting subnetwork, the proposed method directly uses RetinexNet [42] as the image relighting subnetwork. The classic Retinex theory models the human color perception which assumes that the observed images can be decomposed into two components: reflectance and illumination channels. Let *S* represent a source image, then it can be denoted by S=R∗I, where *R* represents reflectance component, *I* represents illumination component, and * represents element-wise multiplication. Here, the loss function of RetinexNet is written as $L_{enh}$. The loss function $L_{enh}$ is formulated as

(4) $${\;L}_{enh}=\;L_{recon}+\;{\lambda_{ir}L}_{ir}+\;{\lambda_{is}L}_{is},$$

where $\lambda_{ir}$ and $\lambda_{is}$ denote the coefficients to balance the reflectance and the illumination. Loss functions $L_{recon}$, $L_{ir}$ and $L_{is}$ represent reconstruction, invariable reflectance, and illumination smoothness functions, respectively.

### 3.5. Pedestrian Detection Subnetwork

In the pedestrian detection subnetwork, we notice that the CornerNet-Saccade has made remarkable achievements in the key-point based object detection algorithm, which uses the Hourglass-54 proposed by [17] as the backbone. Therefore, we design a new Hourglass backbone network, named “SCSAB-Hourglass”, which uses the proposed SCSAB as basic block.

In addition, we propose a lightweight backbone network by pruning SCSAB-Hourglass and named it “SCSAB-HourglassLite”. We apply the focal loss with α=2 and β=4. Here, let $p_{aij}$ be the score at the position $\left(i,j\right)$ for pedestrian in the input image, and let $y_{aij}$ be the ground-truth. Then, we have

(5) $$L_{det}\;=\;\frac{-1}{N}\;\sum_{c=1}^{C}\;\sum_{i=1}^{H}\;\sum_{j=1}^{W}\left\{\begin{array}{c} \;\;{\left(1\;-\;P\right)}^{\alpha }\;\log\left(P\right)\;\;\;\;\;if\left(Y\right)\;=\;1\\ \;\;{\left(1\;-\;Y\right)}^{\beta }\;{\left(P\right)}^{\alpha }\log\left(1\;-\;P\right)\;\;\;otherwise \end{array}\right.,$$

where *N* is the number of pedestrians in an image. *C*, *H*, and *W* represent the number of channels, height, and width, respectively, from input. α and β are the hyperparameters to control the contribution of each point. *P* and *Y* represent $p_{aij}$ and $y_{aij}$, respectively.

When the input image passes through the convolutional layer, the size of the output is usually smaller than the input image. Therefore, a pedestrian position $\left(x,y\right)$ in the image is mapped to the position $\left(\left\lfloor \;\frac{x}{n}\;\right\rfloor ,\left\lfloor \;\frac{y}{n}\;\right\rfloor \right)$ in the heatmaps, where *n* is the downsampling factor. When we remap the positions from the heatmaps to the original size input image, some accuracy may be lost. To solve this issue, we predict the position offsets to adjust the corner positions marginally before remapping them to the original input image.

(6) $$o_{z}=\left(\frac{x_{z}}{n}-\;\left\lfloor \;\frac{x_{z}}{n}\;\right\rfloor ,\frac{y_{z}}{n}-\;\left\lfloor \frac{y_{z}}{n}\right\rfloor \right),$$

where $o_{z}$ is the value of offset, and $x_{z}$ and $y_{z}$ are the *x* and *y* coordinates for corner *z*. For training, we apply the smooth $L_{1}$ loss as the offset loss function, and mark this loss function as $L_{off}$. The details of this function are elaborated in Section 2 of [2]. There exists multiple pedestrians in an image, and thus multiple top-left and bottom-right corners can be detected. We need to determine if a pair of the top-left corner and bottom-right corner is from the same pedestrian. Let $e_{t_{m}}$ be the top-left corner of pedestrian *m* and $e_{b_{m}}$ for the bottom-right corner. As in [43], we use the “pull” and “push” loss to group the corners and separate the corners:

(7) $$L_{pull}=\frac{1}{N}\sum_{m=1}^{N}\left[{\left[e_{t_{m}}-e_{m}\right]}^{2}+{\left[e_{b_{m}}-e_{m}\right]}^{2}\right],$$

(8) $$L_{push}\;=\;\frac{1}{N\;\left(N\;-\;1\right)}\;\sum_{m=1}^{N}\;\sum_{\begin{array}{c} j=1\\ j\neq m \end{array}}^{N}\;\max\left(0,\;1-\;\left|e_{m}\;-\;e_{j}\right|\right),$$

where $e_{m}$ is the average of $e_{t_{m}}$ and $e_{b_{m}}$.

### 3.6. Feature-Level Multi-Task Fusion Learning Module

In this subsection, we introduce how the feature-level multi-task fusion learning module to fuse and share the features of image relighting subnetwork and pedestrian detection subnetwork. The proposed module is shown in Figure 2.

In the feature-level multi-task fusion learning module, we fuse the features $a_{1}^{i}$ and $a_{2}^{i}$ in image relighting subnetwork to the features $b_{1}^{i}$ and $b_{2}^{i}$ in pedestrian detection subnetwork respectively in the $i^{th}$ iteration, and the two fused features are marked as $F_{1}^{i}$ and $F_{2}^{i}$. The feature $a_{1}^{i}$ and feature $a_{2}^{i}$ are from the first 3×3 convolutional layer and the third last 3×3 convolutional layer in Enhance-Net, respectively. The feature $b_{1}^{i}$ and feature $b_{2}^{i}$ are from the second last SCSAB and the first SCSAB in SCSAB-Hourglass, respectively. Meanwhile, the sizes of these features are the same. The $F_{1}^{i}$ and $F_{2}^{i}$ are formulated as

(9) $$F_{1}^{i}=\left(a_{1}^{i}+b_{1}^{i}\right)/2,\;\;\;\;\;F_{2}^{i}=\left(a_{2}^{i}+b_{2}^{i}\right)/2.$$

Then, $F_{1}^{i}$ and $F_{2}^{i}$ with a sigmoid activation function are marked as $sigmoid(F_{1}^{i})$ and $sigmoid(F_{2}^{i})$. In the ${\left(i+1\right)}^{th}$ iteration, $sigmoid(F_{1}^{i})$ is element-wise product by $a_{1}^{i+1}$ and $b_{1}^{i+1}$, respectively, as the input of the second 3 × 3 convolution layer of Enhance-Net and the last SCSAB of the SCSAB-Hourglass, the inputted features are marked as $I_{a_{1}}^{i+1}$ and $I_{b_{1}}^{i+1}$, which are formulated as

(10) $$I_{a_{1}}^{i+1}=\left[a_{1}^{i+1}\times sigmoid\left(F_{1}^{i}\right)\right],$$

(11) $$I_{b_{1}}^{i+1}=\left[b_{1}^{i+1}\times sigmoid\left(F_{1}^{i}\right)\right].$$

The same fusion and sharing method is used as the input of the second last 3 × 3 convolution layer in Enhance-Net and the second SCSAB in SCSAB-Hourglass. The inputted features are marked as $I_{a_{2}}^{i+1}$ and $I_{b_{2}}^{i+1}$, which are formulated as

(12) $$I_{a_{2}}^{i+1}=\left[a_{2}^{i+1}\times sigmoid\left(F_{2}^{i}\right)\right],$$

(13) $$I_{b_{2}}^{i+1}=\left[b_{2}^{i+1}\times sigmoid\left(F_{2}^{i}\right)\right].$$

Finally, the detection network training loss function is formulated as

(14) $$L=L_{det}+\delta L_{pull}+\eta L_{push}+\gamma L_{off}+\zeta L_{enh},$$

where δ, η, and γ are the weights for the pull, push, and offset loss, respectively, and ζ is the weight for the light enhancement loss. We set δ and η to 0.1, γ to 1, and ζ to 0.05.

## 4. Experiments and Discussion

### 4.1. Dataset and Implementation Details

The performance of SPMF is evaluated on the CityPersons dataset [44]. The CityPersons is a publicly available large-scale pedestrian detection dataset that contains 2975 images and approximately 20,000 annotated pedestrian instances in training subset. We use the CityPersons validation dataset as our testing subset, which contains 500 images. Because SPMF requires paired low/normal illumination images for training, we need to render low-light images from normal illumination images in the existing CityPersons dataset. At the same time, same operation of rendering low-light images is used to generate testing subset. Our experiments use the RGB spatial brightness adjustment algorithm to generate low-light images. For example, if the current pixel is (50, 100, 200) and the coefficient of adjustment is 1.1, the adjusted pixel is (55, 110, 220). This paper uses low-light images with an adjustment coefficient of 0.8.

The standard Caltech evaluation: log average Miss Rate over False Positive Per Image (FPPI) range of $\left[10^{-2},10^{0}\right]$ (denoted as ${MR}^{-2}$) [45] is used as the evaluation metric for pedestrian detection algorithm. The lower value of ${MR}^{-2}$, the better performance of the algorithm. We use IoU (Intersection over Union) as the threshold for the evaluation metric, with values of 0.5, 0.75, and 0.5:0.95 (0.5:0.95 means average ${MR}^{-2}$ over different IoU thresholds, from 0.5 to 0.95, step 0.05), respectively, to evaluate the performance of different algorithms.

SPMF is implemented in the Pytorch, with 3 RTX 2080Ti GPUs for training. A mini-batch contains 8 images per GPU. The Adam solver is applied. For the image relighting and pedestrian detection subnetworks, we use the end-to-end alternately training method. First of all, we train two subnetworks, respectively. Then the two trained models are used as pretrained models to train 100 epochs by feature-level multi-task fusion learning module.

### 4.2. Ablation Experiments

In this subsection, we conduct the ablation studies on the low-light CityPersons testing dataset to demonstrate the effectiveness of the proposed backbones.

Comparing with CornerNet-Saccade, which uses Hourglass-54 as the backbone network, we design a new hourglass backbone network in SPMF. The novel hourglass network uses proposed SCSAB as the basic block, which is named “SCSAB-Hourglass”. In addition, we propose a more lightweight backbone than “SCSAB-Hourglass” and name it “SCSAB-HourglassLite”, which is reduced the parameters of model greatly by pruning SCSAB-Hourglass. We name the method using SCSAB-Hourglass backbone as the SPMF, and the method using SCSAB-HourglassLite backbone as SPMF-Lite.

Table 1 shows the parameters of different backbone networks, we can find that comparing with the Hourglass-54, the number of parameters of SCSAB-Hourglass was reduced by about 14%. Meanwhile, the parameters of the proposed SCSAB-HourglassLite have been greatly reduced by about 50% comparing with SCSAB-Hourglass. From the experiment results shown in Table 2, the proposed SCSAB indeed brings an obvious gain in performance. Even we use the SCSAB-HourglassLite backbone network, the detection performance does not decrease much, especially when IoU = 0.5, the ${MR}^{-2}$ of SCSAB-HourglassLite actually is lower than that of SCSAB-Hourglass. This confirms the role of new backbones of detecting network.

### 4.3. Comparison with State-of-the-Art

In this subsection, we select some excellent pedestrian detection or object detection methods as: CSP [15], ALFNet [13], CenterNet [14], CornerNet [16], and CornerNet-Saccade [17]. ALFNet is the most representative algorithm using anchor boxes in pedestrian detection, the CSP and CenterNet are the best algorithms using the central point based method in pedestrian detection and object detection, respectively. Meanwhile, CornerNet and CornerNet-Saccade are the representative algorithms for the point of corner-based method in object detection. The work in [18] takes into account the low light environment, but it learns to extract both RGB images feature and thermal images feature. Therefore, it is not fair to compare with this method.

For fair comparison, two different testing modes are used in testing: end-to-end testing mode and cascade testing mode. In end-to-end testing mode, we use same training dataset to train every selected state-of-the-art, from this point, because both low/normal light images are fed into networks, all these pedestrian detection networks has abilities to deal with low-light images.

#### 4.3.1. End-to-End Testing Mode

**Figure 5:**
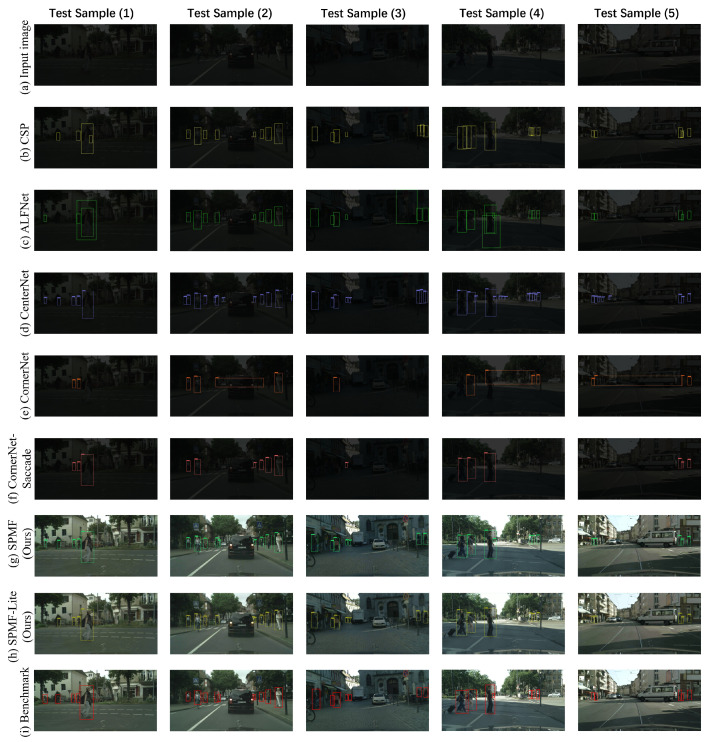
Visual results (end-to-end testing mode) from the different detection methods. Five images from the low-light CityPersons testing set is selected as the samples to show the detected results (with marked bounding box in different color).

Table 3 list the results for end-to-end testing mode, it is obviously that SPMF is superior to these state-of-the-art algorithms. In terms of visual performance, Figure 5 shows the detection results of different methods in five representative samples. Row (a) is the input images, and row (i) is the ground-truth as the benchmark for each detection algorithms. In these visual results, rows (b–d) are from the outputs of CSP, ALFNet, and CenterNet, respectively, in five test samples. Row (b) has missed detection in Test Sample (1)–(3) and has false detection in Test Sample (1)–(5). In the results of row (c), Test Sample (2) appears missed detection, Test Sample (4) has some false detection, and Test Samples (1) and (3) not only have missed detection, but also have false detection. Although all pedestrians are detected in row (d) except Test Sample (2), there are lots of false detections in Test Samples (2)–(5). Rows (e,f) are the test results for CornerNet and CornerNet-Saccade respectively. We can find that row (e) has many missed detection and false detection in these test sample, and row (f) has many missed detection. In the above five algorithms, we find that CornerNet achieves a very poor visual detection results. The main reason for this situation may be that the CornerNet needs 10 GPUs (10 Titan X (PASCAL), total of 120 GB) and uses a batch size of 49 to train, but we do not have such huge computing resources. Under the same experimental conditions (3 RTX 2080Ti GPUs, total of 33 GB), we train the CornerNet with a batch size of 9. The visual results of our models are shown in rows (g,h), intuitively, SPMF and SPMF-Lite achieve good detecting results and with high visual performance. In Test Sample (2), two pedestrians are severely obscured, and it is difficult to see the two obscured pedestrians in an intuitive visual experience. However, SPMF and SPMF-Lite can still detect partially severely obscured pedestrians, and other state-of-the-art cannot detect these, which also proves the proposed methods having high performance.

#### 4.3.2. Cascade Testing Mode

**Figure 6:**
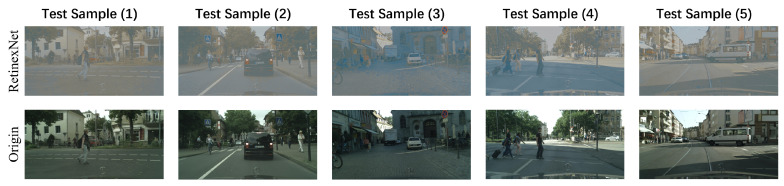
The results of five selected test samples passing through the RetinexNet network.

In cascade testing mode, we separately train relighting and detecting of two tasks, where RetinexNet is used for relighting task and the other state-of-the-art pedestrian detection algorithms are used for detecting task. Thus, in this setting, it is more fair to compare SPMF with two cascaded tasks. Figure 6 shows the outputs from the RetinexNet for five selected test samples in low-light CityPersons testing set, we can find that comparing with the original images, the images output by RetinexNet has caused some distortion of texture information and loss of color information on the basis of restoring image brightness.

**Figure 7:**
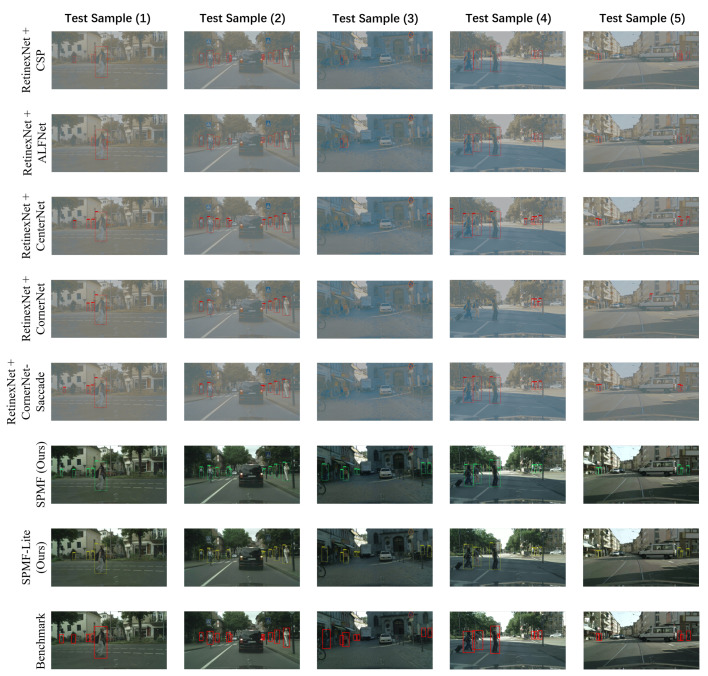
Visual results (cascade testing mode) from the different detection methods. Five images from the low-light CityPersons testing set is selected as the samples to show the detected results.

The results of cascade testing mode are shown in Table 4, and we can see that even if we use the images after relighting for testing these state-of-the-art algorithms, SPMF still achieves awesome performance, which proves the effectiveness of proposed multi-task learning method. The visual results of cascade testing mode are shown in Figure 7, intuitively, the detecting performance of these state-of-the-art algorithms in the cascade testing mode are still unsatisfactory. Especially in the test sample (3), these algorithms have achieved very poor detection results, even two algorithms (CornerNet and CornerNet-Saccade) can not detect pedestrians. The reason for this situation may be that comparing with other test samples, test sample (3) had more distortion of texture information and loss of color information when it is relighted by RetinexNet. In contrast, SPMF can relight image with high visual quality, which is also one of the key factors for SPMF to achieve high detection results.

#### 4.3.3. Visual Results on Real-World Images

**Figure 8:**
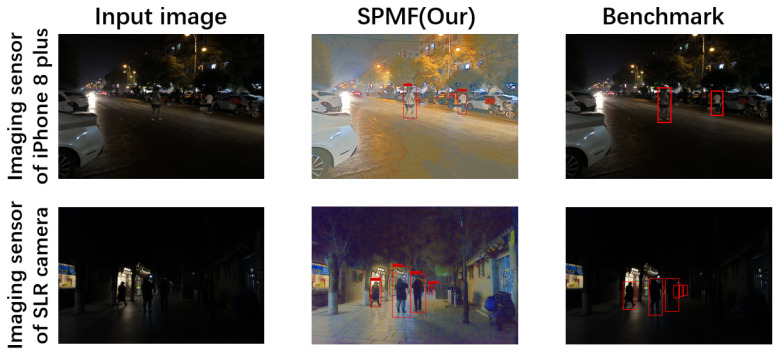
The visual results on real-world images.

In this subsection, we use two different imaging sensors, the SLR (Single Lens Reflex) camera and the iPhone 8 Plus, to obtain real-world low-light images and test the proposed method. Figure 8 shows the visual results of the proposed method on real-world low-light images. The first line shows the visual results obtained on the iPhone 8 Plus imaging sensor, and the second line shows the visual results obtained on the SLR imaging sensor. The pedestrians in the benchmark images are manually labeled.

We can intuitively see that our method not only achieves good detection performance, but it is also satisfactory for the relighting image, and can better recover pedestrians in real-world low-light images. These results further show that even if the proposed method is trained on low-light dataset in non-real-world scenes, the proposed method still maintains high reliability on low-light images in real world scenes. It also provides the reference and inspiration for the feasibility research of real-world scenarios in the future.

## 5. Conclusions

In this paper, a novel multi-task learning method is proposed, which uses a feature-level fusion and sharing mechanism to fuse the features from the image relighting and pedestrian detection networks, and then the fused features are learned for each network to boost relighting and detecting performances together. Meanwhile, we introduce a novel Self-Calibrated Split Attention Block as the basic block of backbone network, which further improves detecting performance. Experimental results show that the proposed multi-task learning method can also effectively improve the performance of pedestrian detection algorithms in the dark. However, our proposed method also has some limitations. The first point is that the method used to obtain low-light dataset cannot simulate the real-world low-light image well. In the later stage, we will add noise and nonlinear pixel transformation in image preprocessing. The second point is that the proposed method does not have high adaptive generalization ability. If the input is regular-lighting images, the image will be seriously damaged after entering the image relighting subnetwork. In the later stage, we will design a switch network to determine whether the input image needs image relighting. In the near future, we will compare the proposed multi-task feature fusing-sharing learning method with other excellent multi-task learning methods in different tasks, and some extension of multi-task learning should be investigated for other vision-based applications, such as person re-identification, semantic segmentation, pose estimation, etc.

## Figures and Tables

**Table 1:** The parameters of different backbone networks.

Backbones	Parameters
Hourglass-54	116.97 M
SCSAB-Hourglass	100.74 M
SCSAB-HourglassLite	50.18 M

**Table 2:** The experimental results of different backbone networks in SPMF.

Backbones	${\mathrm{MR}}^{-2}$(%)		
	IoU = 0.5	IoU = 0.75	IoU = 0.5:0.95
Hourglass-54	15.9	62.3	64.8
SCSAB-Hourglass	12.5	56.6	58.8
SCSAB-HourglassLite	11.7	57.2	60.4

**Table 3:** Comparison with the state-of-the-art on low-light CityPersons (End-to-end testing mode).

Methods	${\mathrm{MR}}^{-2}$(%)		
	IoU = 0.5	IoU = 0.75	IoU = 0.5:0.95
CSP [15]	24.8	64.4	59.9
ALFNet [13]	35.6	62.5	62.6
CenterNet [14]	22.8	64.7	62.7
CornerNet [16]	40.2	79.9	78.0
CornerNet-Saccade [17]	35.3	76.6	77.8
SPMF (Ours)	12.5	56.6	58.8
SPMF-Lite (Ours)	11.7	57.2	60.4

**Table 4:** Comparison with the state of the arts on low-light CityPersons (Cascade testing mode).

Methods	${\mathrm{MR}}^{-2}$(%)		
	IoU = 0.5	IoU = 0.75	IoU = 0.5:0.95
RetinexNet+CSP [15]	31.5	56.7	59.3
RetinexNet+ALFNet [13]	33.5	59.6	60.1
RetinexNet+CenterNet [14]	24.1	65.9	64.3
RetinexNet+CornerNet [16]	39.9	77.9	76.5
RetinexNet+CornerNet-Saccade [17]	29.9	71.4	73.1
SPMF (Ours)	12.5	56.6	58.8
SPMF-Lite (Ours)	11.7	57.2	60.4
